# Supplementary material for: Hypoxic-ischemic brain injury in neonatal mice sequentially recruits neutrophils with dichotomous phenotype and function
Source: Nat Commun. 2025 Nov 3;16:9696. doi: 10.1038/s41467-025-65517-1 (PMC12583616; doi:10.1038/s41467-025-65517-1)
Supplement: Supplementary file 1 — Supplementary Information [file 41467_2025_65517_MOESM1_ESM.pdf]

Supplementary Material

Hypoxic-ischemic brain injury in neonatal mice sequentially recruits neutrophils with dichotomous phenotype and function

Mathis Richter[1,2,3 *], Eva Diesterbeck[1,2 *], Ekaterina Pylaeva[4,5], Nicole Labusek[1,2], Christian Köster[1,2], Dennis Nagel[6], Laura Karsch[6], Alexa Josephine Fischer[1,2], Marah Sous[1,2], Marcel Jung[6], Raphael Chevre[3], Nina Hagemann[2,7], Erik Axel Andersson[8,9], C. Joakim Ek[9], Vikramjeet Singh[6], Dirk M. Hermann[2,7], Matthias Gunzer[6,10], Jadwiga Jablonska[4,5], Ursula Felderhoff-Müser[1,2], Ivo Bendix[1,2], Oliver Soehnlein[3], Josephine Herz[1,2]

[1] Department of Pediatrics I, Neonatology & Experimental Perinatal Neurosciences, University Hospital Essen, University Duisburg-Essen, Germany

[2] Center for Translational Neuro- and Behavioral Sciences (C-TNBS), University Hospital Essen, University Duisburg-Essen, Germany

[3] Institute of Experimental Pathology (ExPat), Center of Molecular Biology of Inflammation (ZMBE), University of Münster, Germany

[4] Department of Otorhinolaryngology, Translational Oncology, University Hospital Essen, University Duisburg-Essen, Germany

[5] German Cancer Consortium (DKTK) partner site Düsseldorf/Essen; Essen, Germany

[6] Institute for Experimental Immunology and Imaging, University Hospital Essen, University Duisburg-Essen, Germany

[7] Department of Neurology, University Hospital Essen, University Duisburg-Essen, Germany

[8] Department of Molecular and Clinical Medicine, Institute of Medicine, Wallenberg Centre for Molecular and Translational Medicine, University of Gothenburg, Sweden

[9] Institute of Neuroscience and Physiology, Sahlgrenska Academy, University of Gothenburg, Sweden

[10] Leibniz-Institut Für Analytische Wissenschaften - ISAS - e.V., Dortmund, Germany

* equally contributing authors

Supplementary Figures: 9

Supplementary Tables: 9

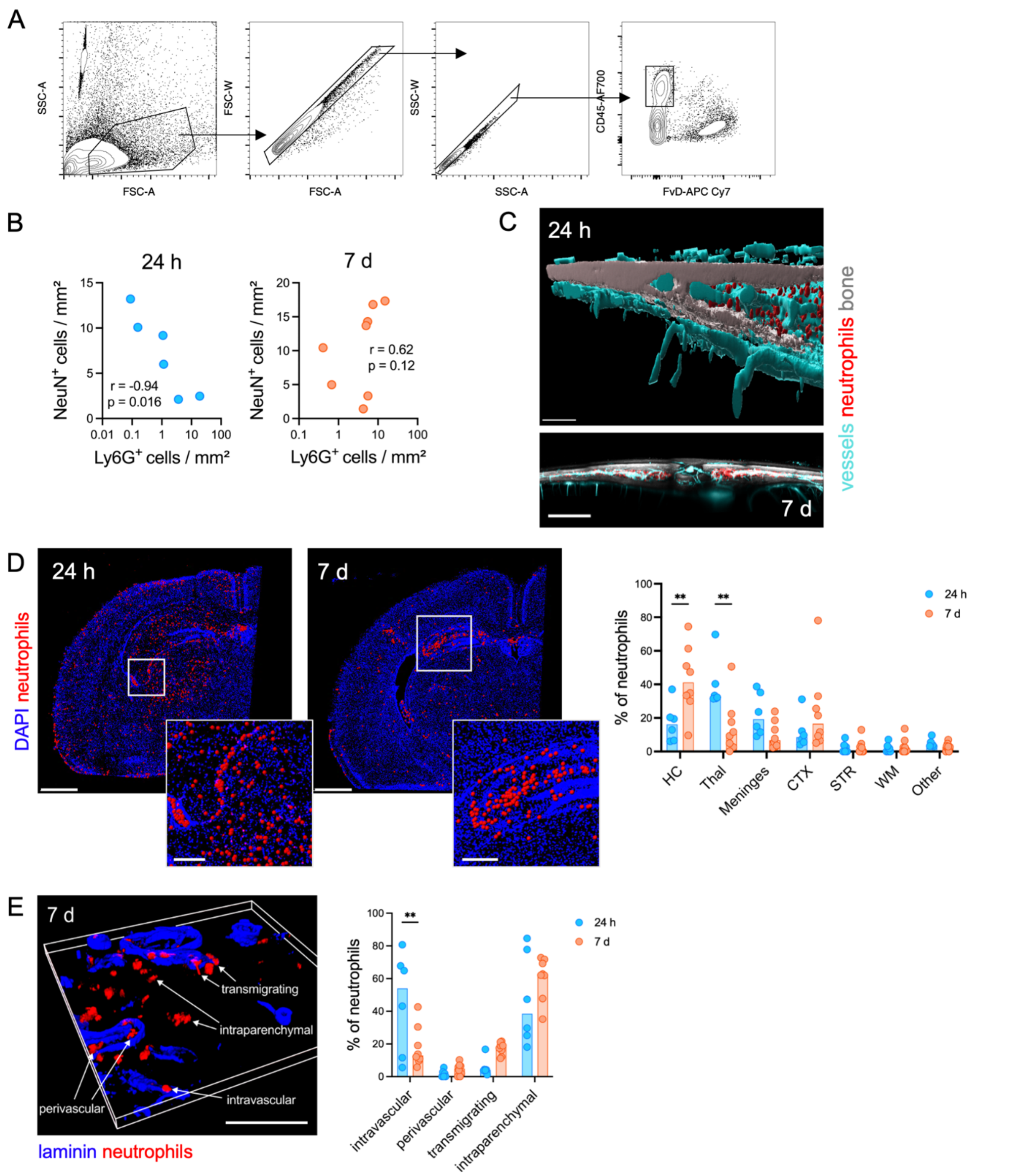

Figure S1: Localization of neutrophils in the injured brain after HI. (A) Flow cytometry pre-gating strategy for Fig 1A. **(B)** Spearman correlation analysis between brain-infiltrated neutrophil cell counts and NeuN$^+$ cells. **(C)** Exemplary two-photon images of neutrophils in the skull bone marrow of neonatal mice, representative for n=2 (24 h) and n=1 (7 d). Scale bars 100 µm (24 h), 200 µm (7 d). **(D)** Regional distribution of neutrophils in the brain 24 h and 7 d after HI. Scale bar overview = 1 mm. Scale bar zoom = 300 µm. Two-way ANOVA followed by Šídák's test. **(E)** Spatial localization of neutrophils in relationship to vessels after HI were quantified according to the classification shown in the exemplary image left, representing an image from the hippocampal region 7 days after HI. Scale bar = 50 µm. Two-way ANOVA followed by Šídák's test. B/D/E: n=6 (24 h), n=8 (7 d). **p<0.01. Exact p values are provided in the source data file. Bar graphs show median values.

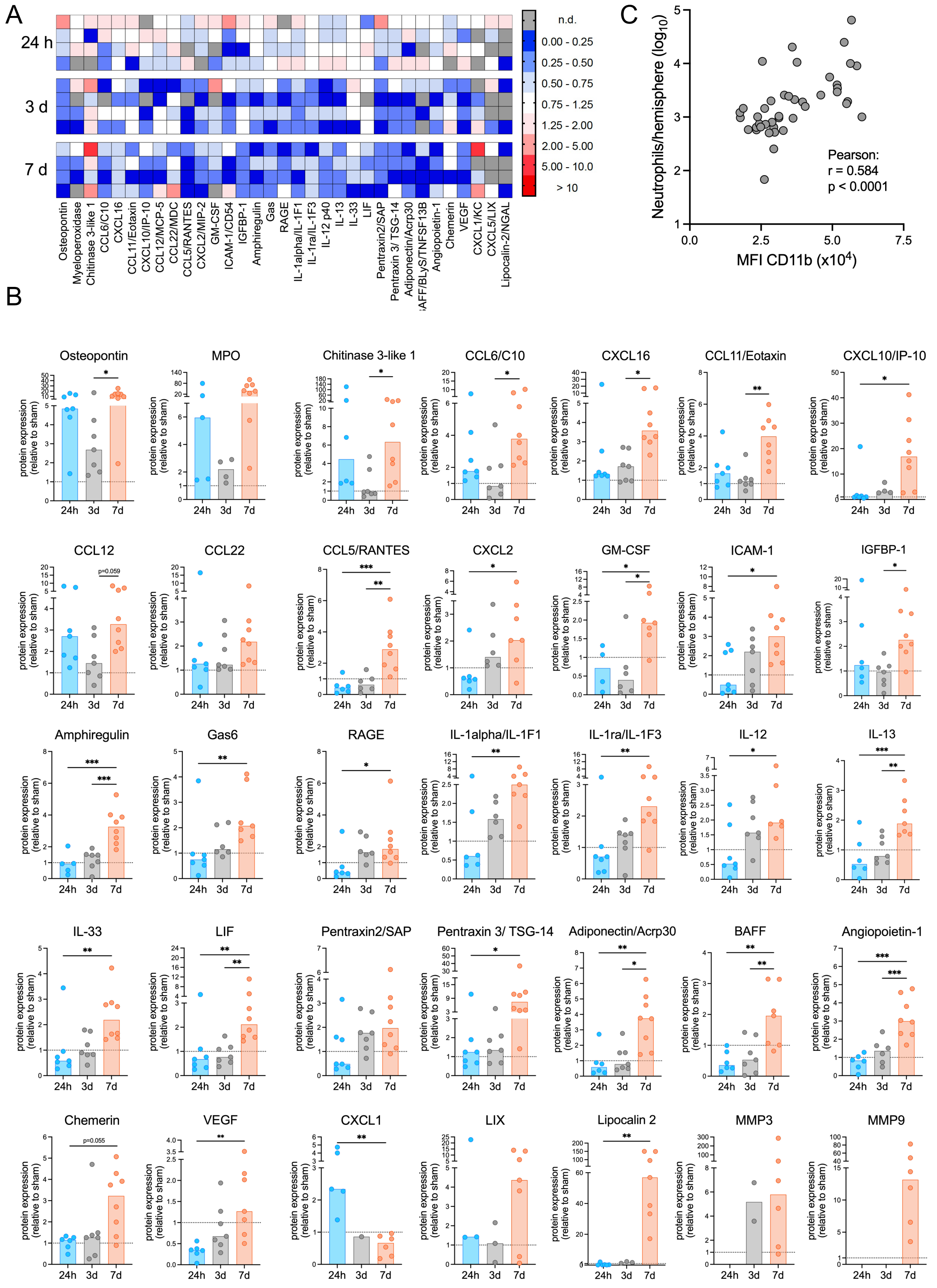

Figure S2: Time course-dependent protein expression of inflammatory, neuroprotective and regenerative proteins. ((A) Expression pattern of proteins analyzed with the Proteome ProfilerTM antibody XL Cytokine array in tissue lysates derived from ipsilateral hemispheres of sham animals. Data were normalized to the mean of 24 h, n=4 per time point, grey values indicate signal intensities below local background. **(B)** Quantification of protein expression in tissue lysates of HI-injured animals from Fig 2A,B; n=7 (24 h/3 d) or n=8 (7 d). Values of HI mice were normalized to the mean of sham animals of the same time point. Differences between time points were analyzed by one way ANOVA or Kruskal

Wallis followed by Šídák's multiple comparisons test or Dunn's test, respectively. Details are provided in the source data file. **(C)** Correlation analysis between brain-infiltrated neutrophil cell counts and CD11b expression in HI-injured animals including all time points (i.e. 12 h, 24 h, 3 d, 7 d, 10 d, n=42). *p<0.05, **p<0.01, ***p<0.001. Exact p values are given in the source data file. Bar graphs show median values.

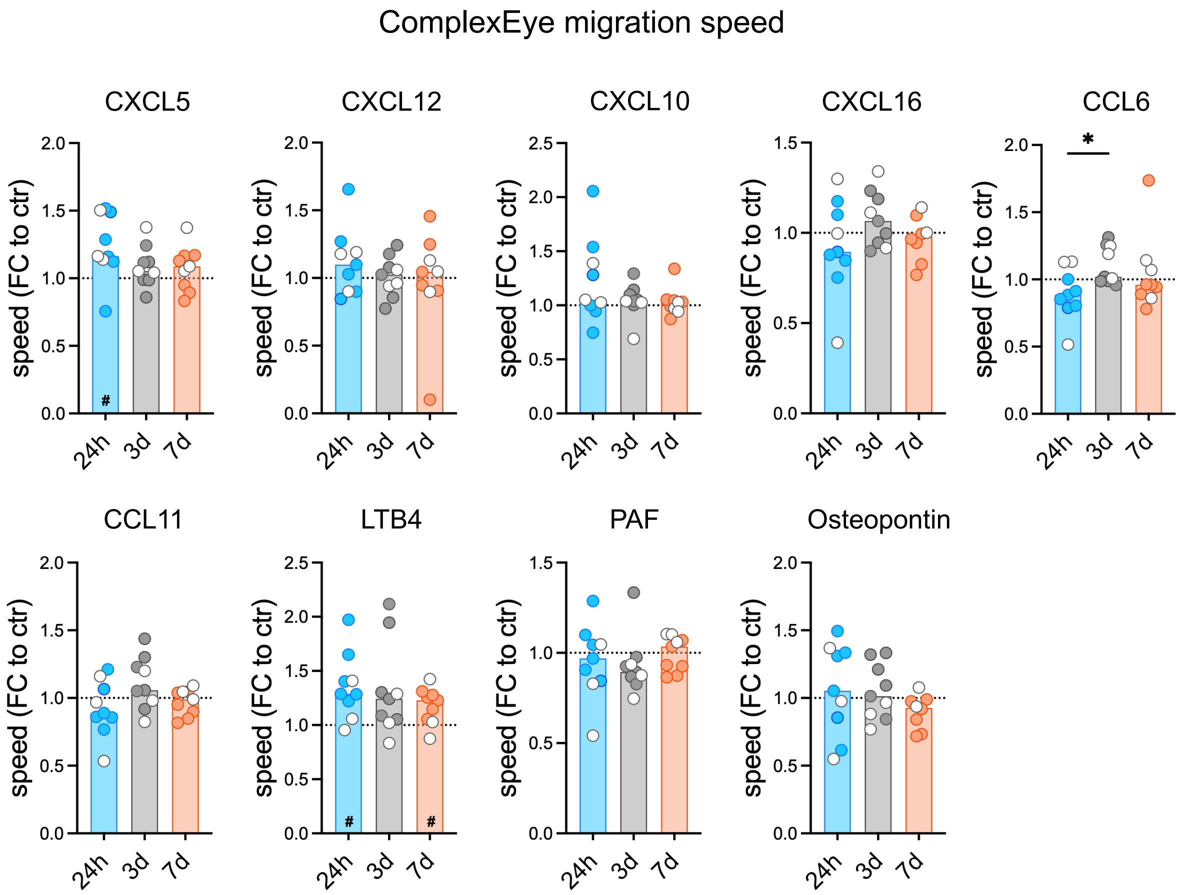

Figure S3: Neutrophil migration response during development. ComplexEye analysis of blood neutrophil migration speed in response to different stimuli normalized to vehicle-treated cells of the same sample. n=9 per time point (n=3 sham, n=6 HI). Open symbols show sham animals. *One-way ANOVA or Kruskal-Wallis followed by Šídák's or Dunn's multiple comparisons test, respectively, with $p<0.05$. # One-sample t tests or Wilcoxon tests against 1 with $p<0.05$. Details on statistical analyses are provided in the source data file. Bar graphs show median values.

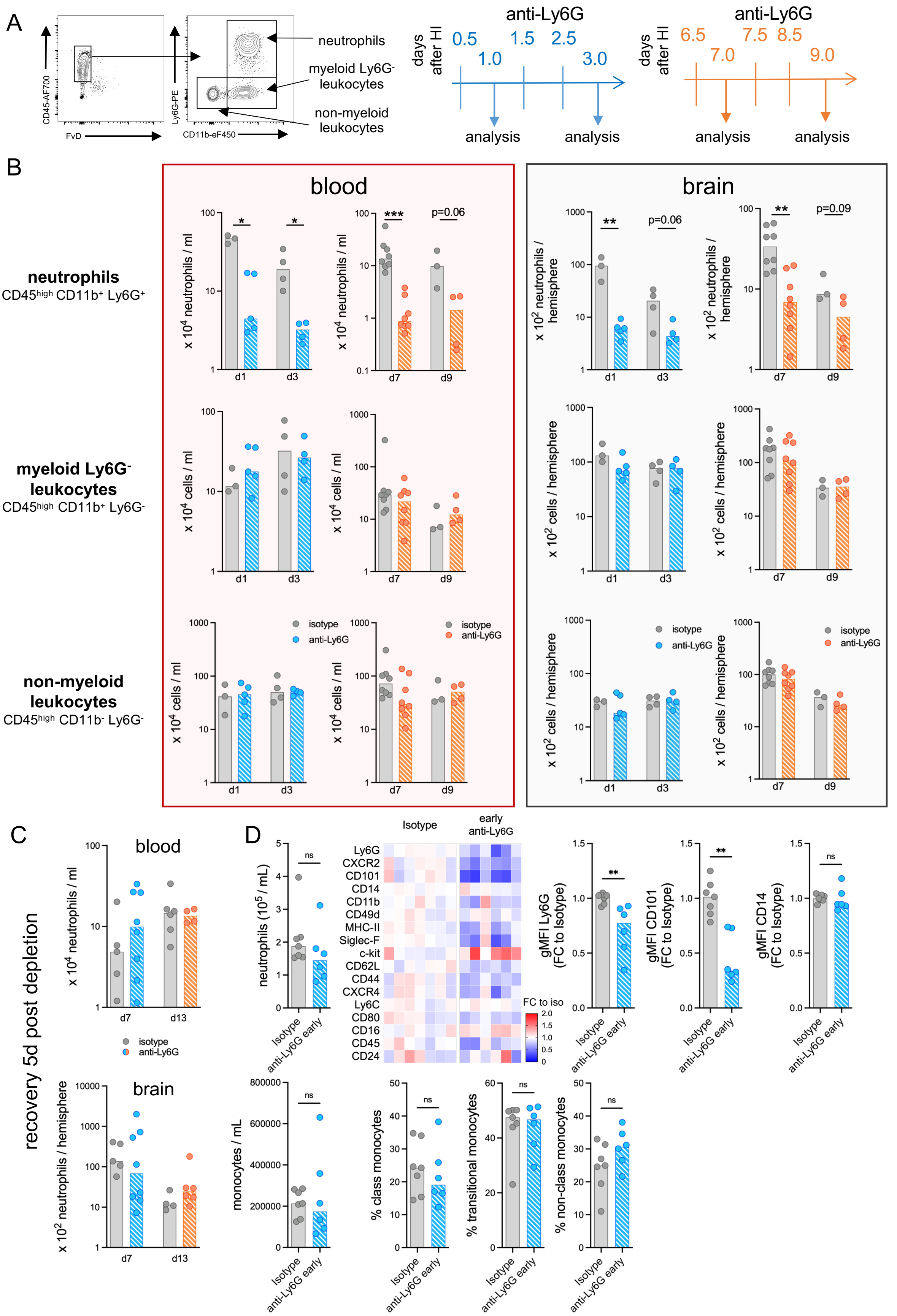

Figure S4: Neutrophil depletion efficiency and recovery. (A) Flow cytometry gating strategy and neutrophil depletion design. Neutrophils and other leukocyte subsets were quantified via flow cytometry in blood and brain samples of HI-injured animals at the indicated time points after HI. Neutrophils were

depleted using an adapted depletion protocol combing anti-Ly6G with anti-rat IgG. Ly6G was stained intracellularly to identify neutrophils after anti-Ly6G injection. **(B)** Quantification of leukocyte subsets in blood and brain at different timepoints as indicated in **(A)**. n=3 (iso d1, iso d9), n=4 (iso d3, anti-Ly6G d3, anti-Ly6G d9), n=5 (anti-Ly6G d1), n=8 (iso & anti-Ly6G d7). **(C)** Recovery of neutrophil numbers 5 days after early or late depletion in blood and brain. n=4 (iso d13), n=5 (iso d7), n=6 (anti-Ly6G d13) n=8 (anti-Ly6G d7). **(D)** Numbers and phenotype of reappearing blood neutrophils ($CD45^+$ $CD11b^+$ $CD115^-$ $Ly6G^+$) at day 10 post HI after early depletion. Cell surface marker expression values of anti-Ly6G-treated animals were normalized to values of isotype mice. Monocyte ($CD45^+$ $CD11b^+$ $CD115^+$ $Ly6G^-$) subsets were identified as classical monocytes ($Ly6C^{high}$ $CD62L^{high}$), transitional monocytes ($Ly6C^{int}$ $CD62L^{low}$) or non-classical monocytes ($Ly6C^{low}$ $CD62L^{low}$). Isotype n=7, anti-Ly6G n=6. *p<0.05, **p<0.01, ***p<0.001, unpaired two- tailed t-tests or Mann Whitney tests. Exact p values are given in the source data file. Bar graphs show median values.

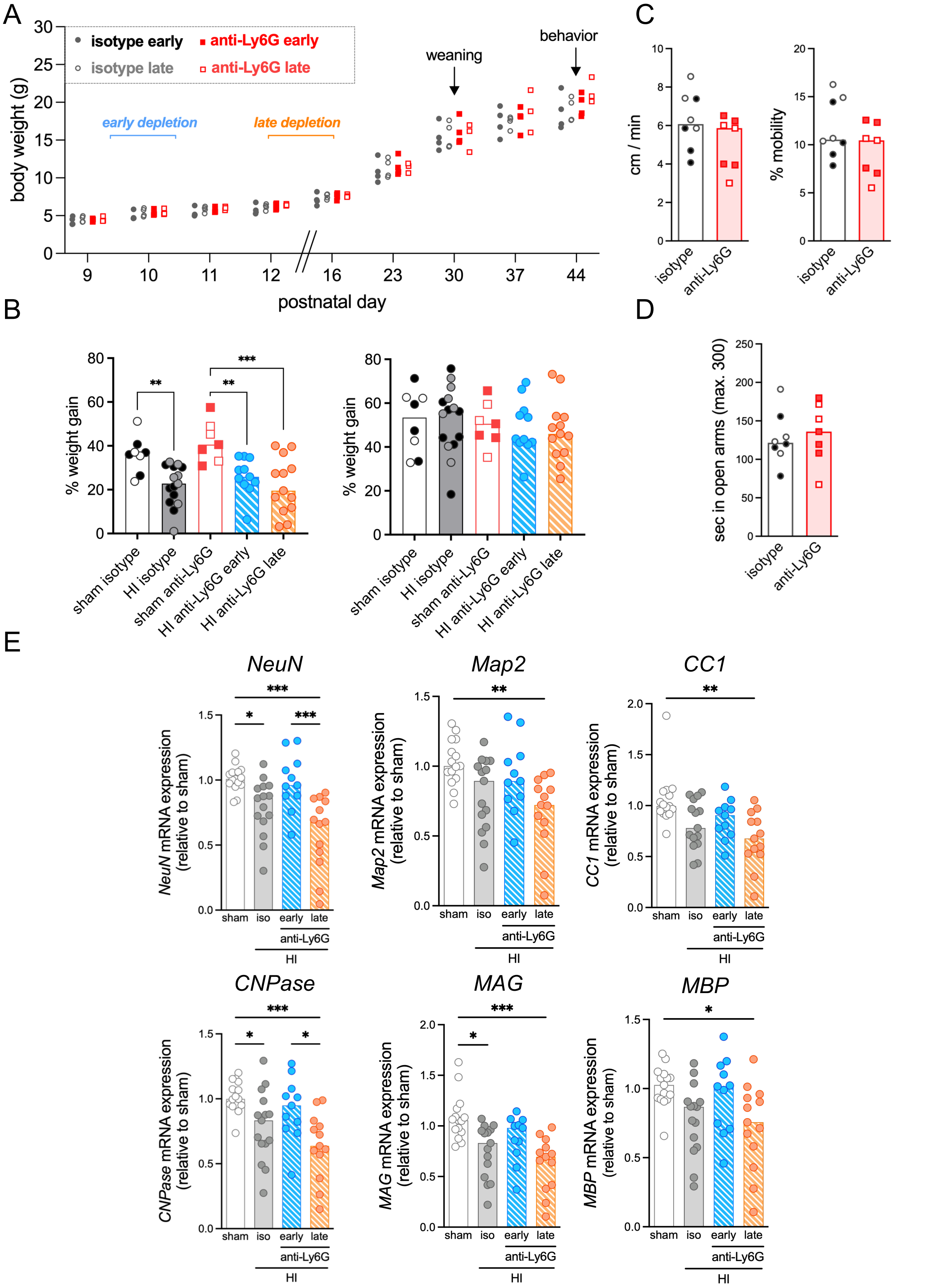

Figure S5: Impact of neutropenia on developmental weight gain and long-term neurological function in sham animals and neuronal/myelination development in HI mice. (A) Developmental weight gain in sham-operated animals after early and late neutrophil depletion and in isotype-treated control animals. **(B)** Percent weight gain between 0-3 days (left) and 7-14 days after HI or sham operation for all experimental groups; one-way ANOVA followed by Holm-Šídák's multiple comparisons

test. Filled symbols in sham isotype, sham-anti- Ly6G, HI-isotype groups indicate mice that received early antibody injection, open symbols show animals with late treatment. **(C, D)** Impact of neutrophil depletion on activity parameters of sham mice analyzed in the open field test by measurement of mean movement velocities and percent time of mobility **(C)** and on anxiety-related behavior assessed in the elevated plus maze test by measurement of the time, the mice spent in the open arms **(D)** 5 weeks after sham operation. Filled symbols and open symbols indicate early and late treatment, respectively. **(E)** mRNA expression of different neuronal (*NeuN, MAP2*) and myelination (*CC1, CPNase, MAG, MBP*) transcripts. n=15 sham (4 isotype early, 4 isotype late, 4 anti-Ly6G early, 3 anti-Ly6G late), n= 15 HI isotype (8 early, 7 late), n=11 HI anti-Ly6G early, n=13 HI anti-Ly6G late. One-way ANOVA or Kruskal-Wallis followed by Šídák's or Dunn's multiple comparisons test, respectively. *$p<0.05$, **$p<0.01$, ***$p<0.001$. Exact p values are given in the source data file. Bar graphs show median values.

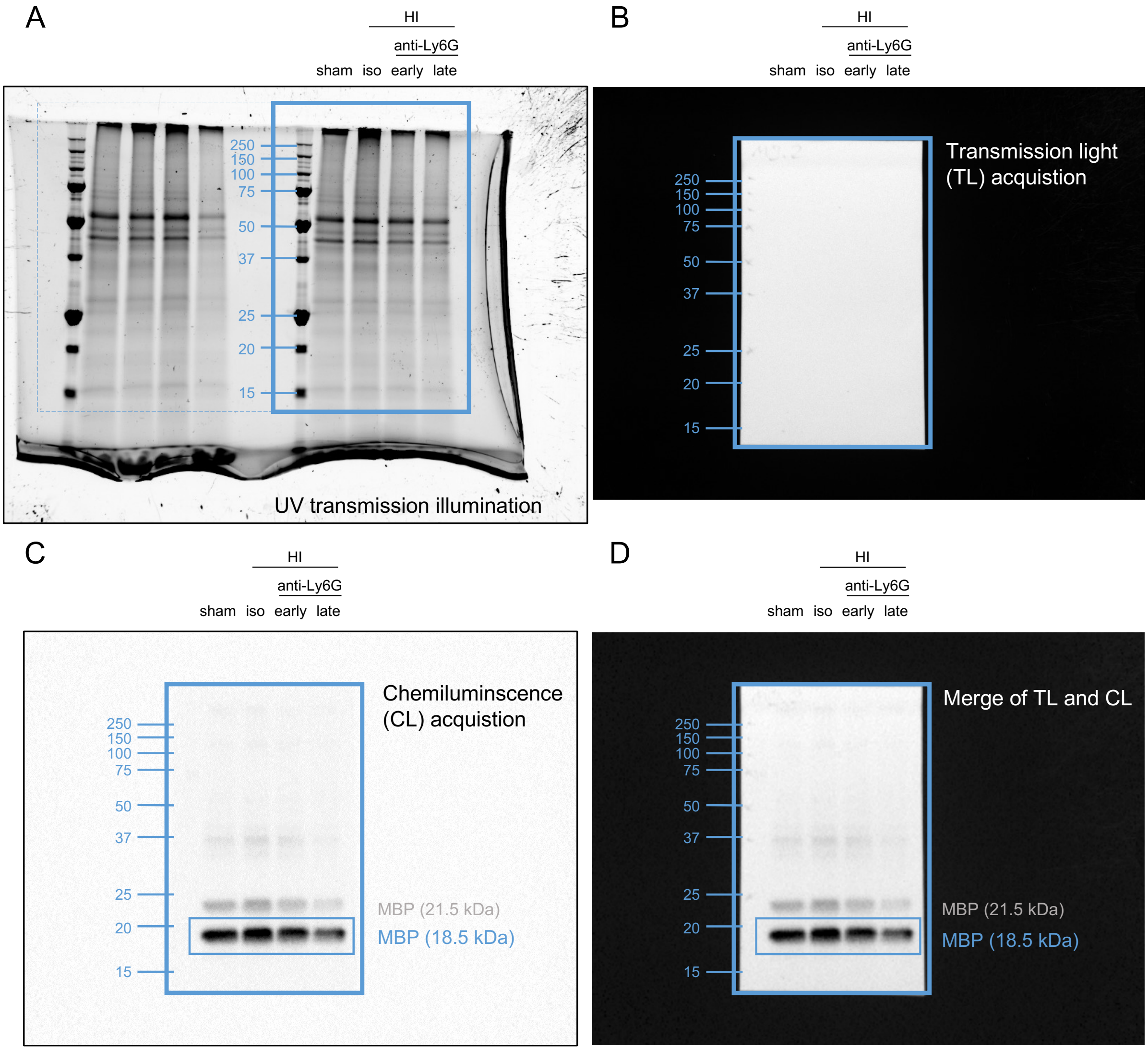

Figure S6: Full length gels and membranes used for representative images. (A) Total protein abundance was visualized after gel electrophoreses on 12.5 % SDS gels via ultraviolet light illumination. The right part of the gel (indicated by the rectangle) was used for western blotting and detection of MBP proteins. **(B)** Pictures of membranes with blotted proteins were first captured with transmission light illumination (TL) to mark major ladder bands. **(C)** Chemiluminescence illumination was used to visualize anti-MBP antibody binding. **(D)** Merged images of **(B)** and **(C)** were used for cropped images shown in Fig. 3H.

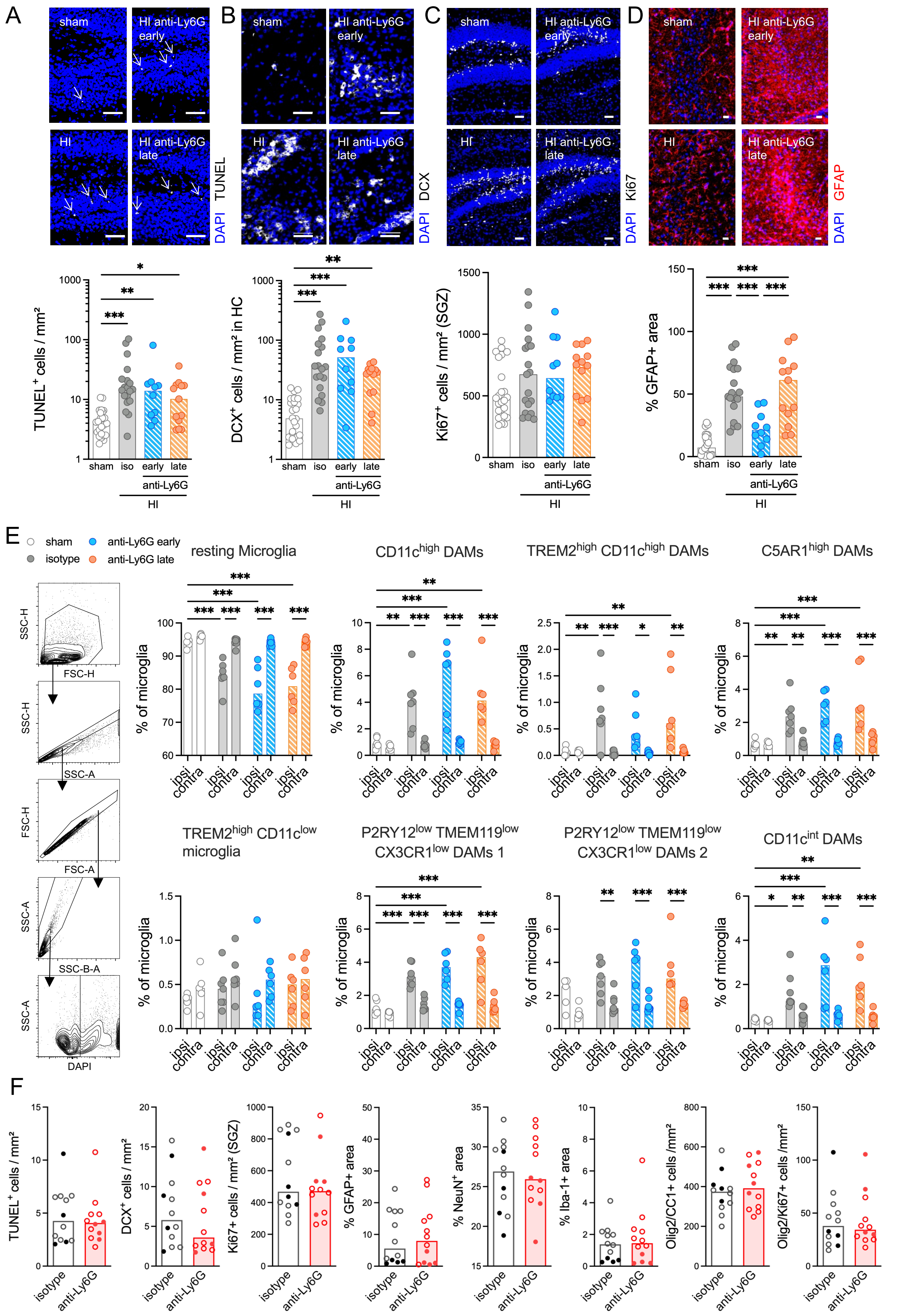

Figure S7: Impact of neutrophil depletion on HI-induced secondary apoptosis, neurogenic response, astrogliosis and myeloid cell diversity. (A) Apoptosis, **(B)** neurogenesis, **(C)** proliferation and **(D)** astrogliosis were evaluated by staining of TUNEL, doublecortin (DCX), Ki67 and GFAP,

respectively. Scale bar: 50 µm. n=12 sham isotype (4 early, 8 late), n=12 sham anti-Ly6G (4 early, 8 late), n=19 HI isotype (8 early, 11 late), n=10 anti-Ly6G early, n=13 HI anti-Ly6G late. One-way ANOVA or Kruskal-Wallis followed by Holm-Sidak's or Dunn’s multiple comparisons test, respectively. Details are given in the source data file. **(E)** Pre-gating strategy and individual data for quantification of macrophage/microglia cell composition using spectral flow cytometry shown in Fig. 4D. n=5 sham (3 isotype (1 early, 2 late), 2 anti-Ly6G (1 early, 1 late), n=7 HI isotype (3 early, 4 late), n=6 HI anti Ly6G early, n=6 HI anti-Ly6G late, two-way ANOVA followed by Šídák's test. **(F)** Effect of isotype- or anti-Ly6G-treatment on sham animals; filled and open symbols indicate early and late treatment, respectively. n=12 sham isotype (4 early, 8 late), n=12 sham anti- Ly6G (4 early, 8 late). *p<0.05, **p<0.01, ***p <0.001. Exact p values are given in the source data file. Bar graphs show median values.

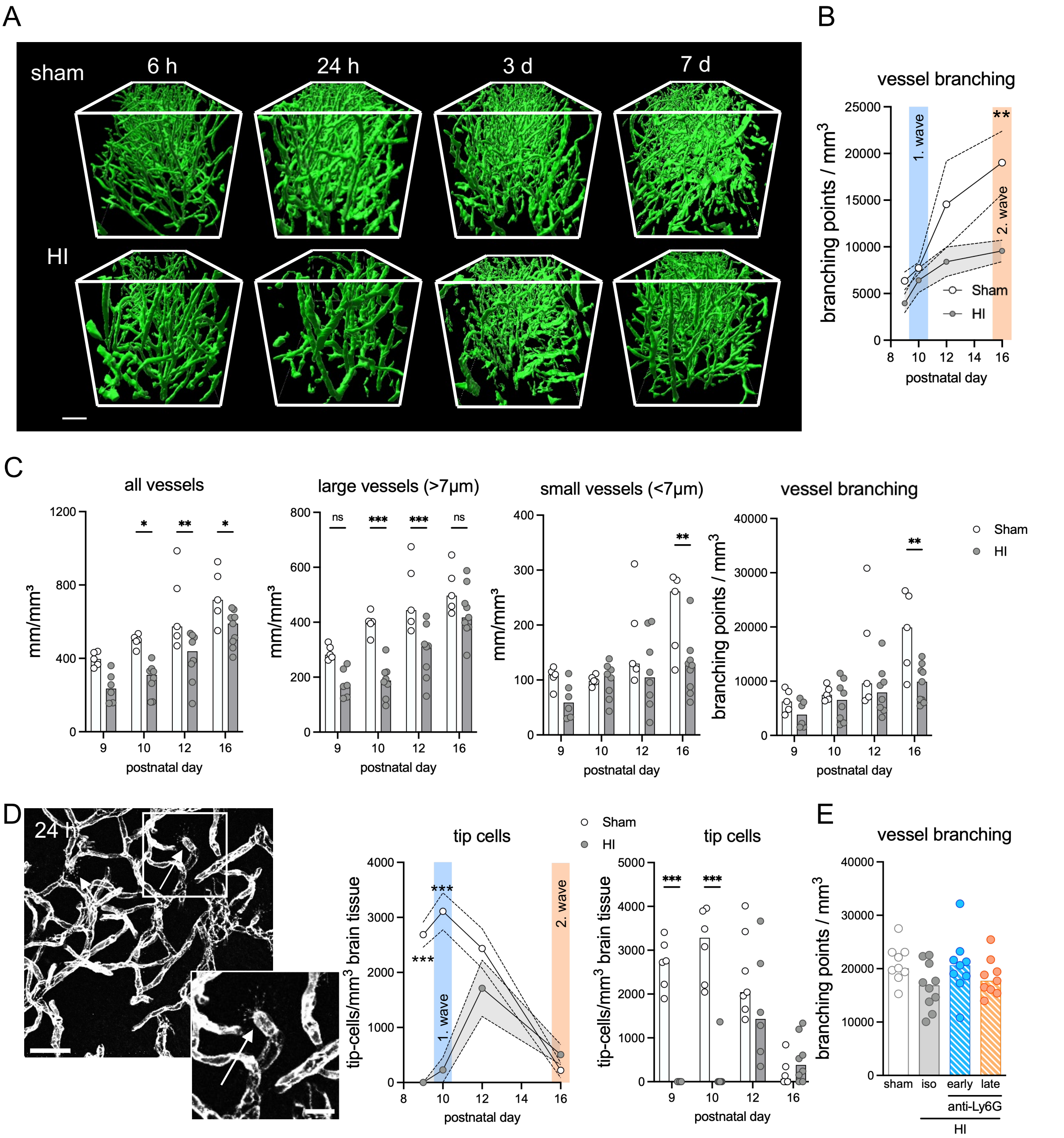

Figure S8: Effect of neonatal HI on vascular development (A) 3D projections of the hippocampal vasculature analyzed by light sheet microscopy between postnatal day 9 and 16 (P9-P16) in the ipsilateral hemisphere of sham and HI mice. Scale bar = 100μm. **(B)** Quantification of branching points. Mean and SEM values are shown form n=5 sham/time point, n=6 P9 HI, n=8 P10/P12 HI, n=9 P16 HI, two-way ANOVA followed by Šídák's test. **(C)** Individual data for quantification of vessel length density of all vessels, large vessels (>7 μm) or small vessels (<7 μm) and branching points. n=5 sham/time point, n=6 P9 HI, n=8 P10/P12 HI, n=9 P16 HI, two-way ANOVA followed by Šídák's test. **(D)** Quantification of tip cells in HI and sham animals. Mean and SEM values or individual data points are shown from n=6 (sham P9/ P10, HI P9/ P10/ P12), n=7 (sham P12), n=8 (HI P16), n=5 (sham P16), two-way ANOVA followed by Šídák's test. Scale bar overview = 50 μm. Scale bar zoom = 20 μm. **(E)** Quantification of branching points 10 days after HI after early and late neutrophil depletion. n=4 sham isotype (2 early, 2 late), n=5 sham anti-Ly6G (2 early, 3 late), n=10 HI isotype (5 early, 5 late), n=9 HI anti-Ly6G early/anti-Ly6G late *p< 0.05, **p<0.01, ***p<0.001. Exact p values are given in the source data file. Bar graphs in B/C/D/E show median values.

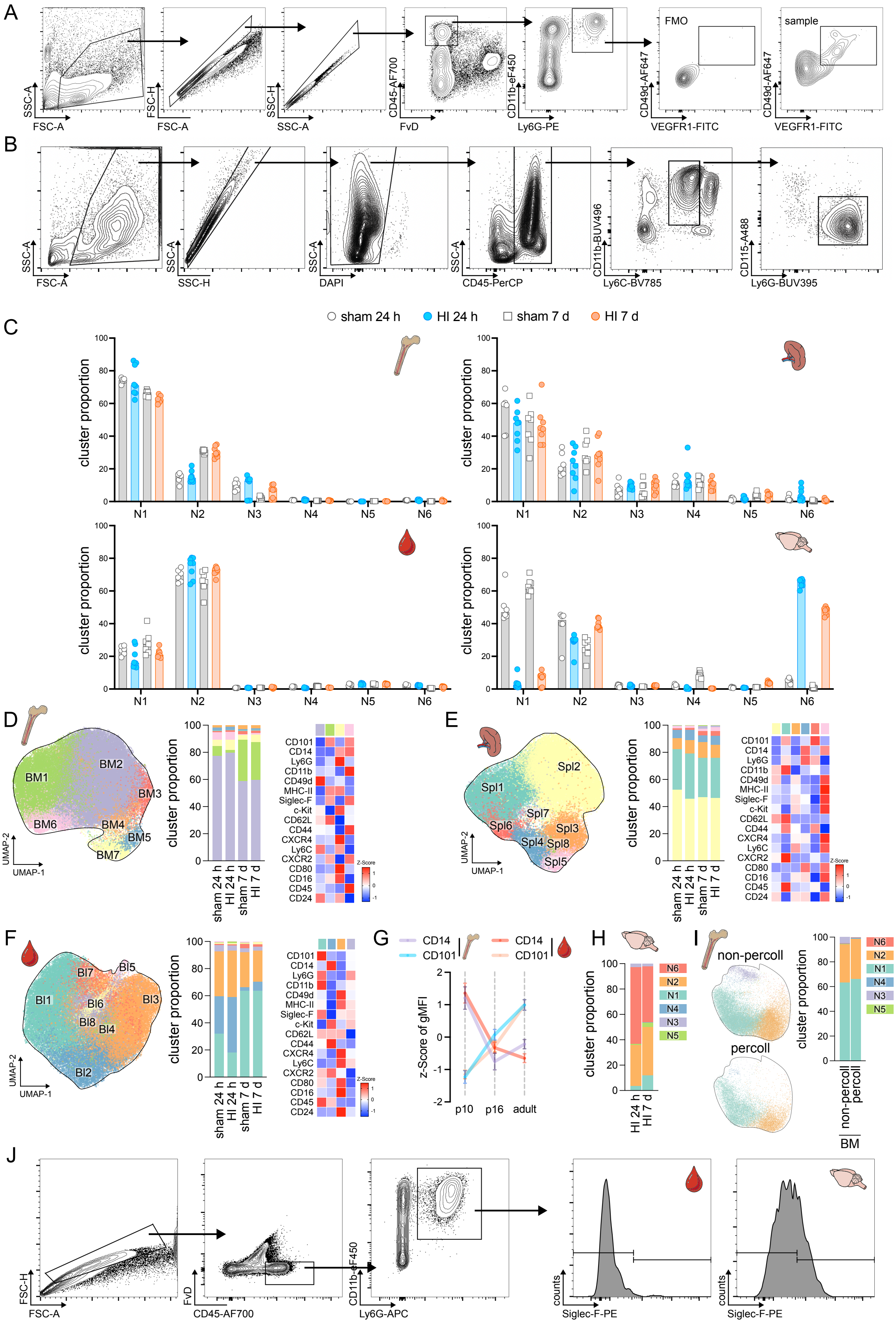

Figure S9: Organ-specific and developmental differences in neutrophil diversity. (A) Gating strategy to identify angiogenic neutrophils. **(B)** Gating strategy to identify neutrophils in spectral flow cytometry analyses. **(C)** Individual relative abundance of neutrophil clusters in different organs. n=6 (sham 24 h), n=7 (sham 7 d), n=8 (HI 24 h/ 7d). **(D-F)** Neutrophils from bone marrow, spleen and blood were analyzed 24 h and 7 d after HI or sham operation. Data of spectral flow cytometry were analyzed

by UMAP dimensional-reduction and FlowSOM clustering in each organ. Mean expression of surface markers of identified organ-specific clusters are displayed for the indicated experimental groups. Clusters with an abundance > 2 % are shown in the heatmaps. **(G)** Z-scores for surface expression of CD101 and CD14 on blood and bone marrow neutrophils of sham animals at p 10 (n=6), p 16 (n=7) and untreated adult 12/13- week-old mice (n=9). Mean and SEM values are shown. **(H)** Cluster distribution of HI brain neutrophils at 24 h and 7 d. **(I)** To assess whether the different cell isolation procedure, used for brain samples, affect neutrophil phenotypes, bone marrow samples were treated with the same protocol, including density gradient centrifugation with 37% percoll and compared to the standard bone marrow cell isolation procedure ("non-percoll"). Identified clusters refer to main Fig 6E, n=6 percoll n=29 non-percoll. **(J)** Sorting strategy to isolate Siglec-F^{low} and Siglec-F^{high} neutrophils. Bar graphs in C show median values.

Supplementary Tables

Table S1: Reports on neuroprotective and neuroregenerative functions of proteins with a selective and significant upregulation 7 days after HI (referring to main Fig. 2A/B and Suppl. Fig. 2B)

Function → Protein ↓	Neuroprotection	Neuroregeneration		
		Synaptic plasticity and neurogenesis	*Oligodendrocyte genesis & myelination*	*Angiogenesis*
**GM-CSF**	Schabitz et al. 2008<br>Shultz et al. 2014	Kruger et al. 2007		
**Amphiregulin**		Falk & Frisen et al. 2002<br>Park et al. 2023		
**Gas6**	Gruber et al. 2014		Goudarzi et al. 2016<br>Jia et al. 2024	
**RAGE**	Shimizu et al. 2020	Lei et al. 2015		
**IL-1alpha/IL-1F1**	Salmeron et al. 2019	Salmeron et al. 2019		
**IL-1ra/IL-1F3**	Pradillo et al. 2012<br>Pinteaux et al. 2006	Pradillo et al. 2017		
**IL-13**	Chen et al. 2022	Li et al. 2023	Chen et al. 2022	Li et al. 2023
**IL-33**	Jiao et al. 2020<br>Yang et al. 2017<br>Korhonen et al. 2015	Wang et al. 2021<br>Rao et al. 2022	Sun et al. 2021<br>Rao et al. 2022	
**LIF**	Davis & Pennypacker 2018<br>Wang et al. 2023	Wang et al. 2023	Davis & Pennypacker 2018<br>Slaets et al. 2022	
**Pentraxin3/TSG-14**	Rajkovic et al. 2018	Fossati et al. 2019<br>Rodriguez-Grande et al. 2015		Rajkovic et al. 2018<br>Rodriguez-Grande et al. 2015
**Adiponectin/Acrp30**	Miao et al. 2021	Bloemer et al. 2019<br>Zhang et al. 2016		
**BAFF/BLyS/TNFSF13B**	Li et al. 2017<br>Wang et al. 2024			
**Angiopoietin-1**		Rosa et al. 2010		Lee et al. 2009
**Chemerin**	Zhang et al. 2019			Bozaoglu et al. 2010<br>Kaur et al. 2010

References
Bloemer J*, et al.* Adiponectin Knockout Mice Display Cognitive and Synaptic Deficits. *Front Endocrinol (Lausanne)* **10**, 819 (2019).
Bozaoglu K*, et al.* Chemerin, a novel adipokine in the regulation of angiogenesis. *J Clin Endocrinol Metab* **95**, 2476-2485 (2010).
Chen D*, et al.* Interleukin 13 promotes long-term recovery after ischemic stroke by inhibiting the activation of STAT3. *J Neuroinflammation* **19**, 112 (2022).
Davis SM, Pennypacker KR. The role of the leukemia inhibitory factor receptor in neuroprotective signaling. *Pharmacol Ther* **183**, 50-57 (2018).
Falk A, Frisen J. Amphiregulin is a mitogen for adult neural stem cells. *J Neurosci Res* **69**, 757-762 (2002).
Fossati G*, et al.* Pentraxin 3 regulates synaptic function by inducing AMPA receptor clustering via ECM remodeling and beta1-integrin. *EMBO J* **38**, (2019).
Goudarzi S, Rivera A, Butt AM, Hafizi S. Gas6 Promotes Oligodendrogenesis and Myelination in the Adult Central Nervous System and After Lysolecithin-Induced Demyelination. *ASN Neuro* **8**, (2016).
Gruber RC*, et al.* Targeted GAS6 delivery to the CNS protects axons from damage during experimental autoimmune encephalomyelitis. *J Neurosci* **34**, 16320-16335 (2014).
Jia J*, et al.* Growth arrest specific protein 6 alleviated white matter injury after experimental ischemic stroke. *J Cereb Blood Flow Metab* **44**, 77-93 (2024).
Jiao M*, et al.* Neuroprotective effect of astrocyte-derived IL-33 in neonatal hypoxic-ischemic brain injury. *J Neuroinflammation* **17**, 251 (2020).

Kaur J, Adya R, Tan BK, Chen J, Randeva HS. Identification of chemerin receptor (ChemR23) in human endothelial cells: chemerin-induced endothelial angiogenesis. *Biochem Biophys Res Commun* **391**, 1762-1768 (2010).

Korhonen P, *et al.* Immunomodulation by interleukin-33 is protective in stroke through modulation of inflammation. *Brain Behav Immun* **49**, 322-336 (2015).

Kruger C, Laage R, Pitzer C, Schabitz WR, Schneider A. The hematopoietic factor GM-CSF (granulocyte-macrophage colony-stimulating factor) promotes neuronal differentiation of adult neural stem cells in vitro. *BMC Neurosci* **8**, 88 (2007).

Lee SW, Kim WJ, Jun HO, Choi YK, Kim KW. Angiopoietin-1 reduces vascular endothelial growth factor-induced brain endothelial permeability via upregulation of ZO-2. *Int J Mol Med* **23**, 279-284 (2009).

Lei C, *et al*. Activation of the high-mobility group box 1 protein-receptor for advanced glycation end-products signaling pathway in rats during neurogenesis after intracerebral hemorrhage. *Stroke* vol. 46,2 (2015)

Li K, Yu W, Cao R, Zhu Z, Zhao G. Microglia-mediated BAFF-BAFFR ligation promotes neuronal survival in brain ischemia injury. *Neuroscience* **363**, 87-96 (2017).

Miao W*, et al.* Adiponectin ameliorates hypoperfusive cognitive deficits by boosting a neuroprotective microglial response. *Prog Neurobiol* **205**, 102125 (2021).

Park JS*, et al.* Amphiregulin normalizes altered circuit connectivity for social dominance of the CRTC3 knockout mouse. *Mol Psychiatry* **28**, 4655-4665 (2023).

Pinteaux E, Rothwell NJ, Boutin H. Neuroprotective actions of endogenous interleukin-1 receptor antagonist (IL-1ra) are mediated by glia. *Glia* **53**, 551-556 (2006).

Pradillo JM*, et al.* Delayed administration of interleukin-1 receptor antagonist reduces ischemic brain damage and inflammation in comorbid rats. *J Cereb Blood Flow Metab* **32**, 1810-1819 (2012).

Pradillo JM*, et al.* Reparative effects of interleukin-1 receptor antagonist in young and aged/co-morbid rodents after cerebral ischemia. *Brain Behav Immun* **61**, 117-126 (2017).

Rajkovic I*, et al.* Pentraxin 3 promotes long-term cerebral blood flow recovery, angiogenesis, and neuronal survival after stroke. *J Mol Med (Berl)* **96**, 1319-1332 (2018).

Rao X*, et al.* Dual roles of interleukin-33 in cognitive function by regulating central nervous system inflammation. *J Transl Med* **20**, 369 (2022).

Rodriguez-Grande B*, et al.* Pentraxin 3 mediates neurogenesis and angiogenesis after cerebral ischaemia. *J Neuroinflammation* **12**, 15 (2015).

Rosa AI, Goncalves J, Cortes L, Bernardino L, Malva JO, Agasse F. The angiogenic factor angiopoietin-1 is a proneurogenic peptide on subventricular zone stem/progenitor cells. *J Neurosci* **30**, 4573-4584 (2010).

Salmeron KE*, et al.* Interleukin 1 alpha administration is neuroprotective and neuro-restorative following experimental ischemic stroke. *J Neuroinflammation* **16**, 222 (2019).

Schabitz WR*, et al.* A neuroprotective function for the hematopoietic protein granulocyte-macrophage colony stimulating factor (GM-CSF). *J Cereb Blood Flow Metab* **28**, 29-43 (2008).

Shimizu Y, *et al*. Neuroprotective Effects of Endogenous Secretory Receptor for Advanced Glycation End-products in Brain Ischemia. *Aging and disease* vol. 11,3 547-558. (2020)

Shultz SR*, et al.* Granulocyte-macrophage colony-stimulating factor is neuroprotective in experimental traumatic brain injury. *J Neurotrauma* **31**, 976-983 (2014).

Slaets H*, et al.* CNS-targeted LIF expression improves therapeutic efficacy and limits autoimmune-mediated demyelination in a model of multiple sclerosis. *Mol Ther* **18**, 684-691 (2010).

Sun Y*, et al.* Therapeutic Opportunities of Interleukin-33 in the Central Nervous System. *Front Immunol* **12**, 654626 (2021).

Wang AA*, et al.* B cell depletion with anti-CD20 promotes neuroprotection in a BAFF-dependent manner in mice and humans. *Sci Transl Med* **16**, eadi0295 (2024).

Wang J*, et al.* Leukemia inhibitory factor, a double-edged sword with therapeutic implications in human diseases. *Mol Ther* **31**, 331-343 (2023).

Wang Y*, et al.* Astrocyte-secreted IL-33 mediates homeostatic synaptic plasticity in the adult hippocampus. *Proc Natl Acad Sci U S A* **118**, (2021).

Yang Y*, et al.* ST2/IL-33-Dependent Microglial Response Limits Acute Ischemic Brain Injury. *J Neurosci* **37**, 4692-4704 (2017).

Zhang D, Wang X, Lu XY. Adiponectin Exerts Neurotrophic Effects on Dendritic Arborization, Spinogenesis, and Neurogenesis of the Dentate Gyrus of Male Mice. *Endocrinology* **157**, 2853-2869 (2016).

Zhang Y*, et al.* Chemerin reverses neurological impairments and ameliorates neuronal apoptosis through ChemR23/CAMKK2/AMPK pathway in neonatal hypoxic-ischemic encephalopathy. *Cell Death Dis* **10**, 97 (2019).

Table S2: Animal group allocation and mortality rates

Readout	Experimental group	Female	Male	Mortality
**Time course neutrophil infiltration, flow cytometry (C57BL/6, 10 litters)**	sham	10	11	
	HI	24	25	4f, 3m
**Time course neutrophil infiltration, light sheet & 2PM microscopy (Catchup^{IVM}, 7 litters)**	sham	7	12	
	HI	20	19	2f, 3m
**Neutrophil localisation - immunohistochemistry (C57BL/6, 2 litters)**	HI 1d	3	3	
	HI 7d	4	5	1m
**Proteome Profiler array (C57BL/6, 6 litters)**	Sham	6	6	
	HI	11	11	
**Complex Eye (C57BL/6, 4 litters)**	Sham	6	3	
	HI	8	10	
**Neutrophil depletion efficiency, flow cytometry (C57BL/6, 5 litters)**	HI Isotype	11	20	2f*
	HI Anti-Ly6G	19	17	1f, 3m*
**Behavioral testing (C57BL/6, 10 litters)**	Sham Isotype early	2	2	
	Sham Isotype late	2	2	
	Sham Anti-Ly6G early	2	2	
	Sham Anti-Ly6G late	1	2	
	HI Isotype early	8	4	4f*
	HI Isotype late	8	5	4f, 2m*
	HI Anti-Ly6G early	7	5	1f*
	HI Anti-Ly6G late	9	7	1f, 2m*
**Immunohistochemistry (C57BL/6, 10 litters)**	Sham Isotype early	2	2	
	Sham Isotype late	4	4	
	Sham Anti-Ly6G early	2	2	
	Sham Anti-Ly6G late	5	3	
	HI Isotype early	5	4	1m*
	HI Isotype late	8	6	3f*
	HI Anti-Ly6G early	6	5	1f*
	HI Anti-Ly6G late	9	6	2m*
**Lightsheet microscopy, vascularisation - time course (C57BL/6, 10 litters)**	Sham	10	10	
	HI	16	17	1f,1m
**Tip cell analyses, immunohistochemistry (C57BL/6, 8 litters)**	Sham	12	12	
	HI	12	14	
**Lightsheet microscopy, vascularisation - anti-Ly6G treatment (C57BL/6, 5 litters)**	Sham Isotype early		2	
	Sham Isotype late	1	1	
	Sham Anti-Ly6G early	1	1	
	Sham Anti-Ly6G late	1	2	
	HI Isotype early	3	3	1f*
	HI Isotype late	3	3	1f*
	HI Anti-Ly6G early	4	5	
	HI Anti-Ly6G late	3	6	
**ROS measurement (C57BL/6, 4 litters)**	HI	11	12	
**Neutrophil sort for aortic ring, mRNA expression and bEND.3 assays (C57BL/6, 18 litters)**	HI	62	52	2f, 4m
	naïve (donor aorta)	1	1	
**Spectral flow cytometry - neutrophils (C57BL/6, 4 litters)**	Sham	4	9	
	HI	9	13	2f, 4m
	adult naïve		9	
**Spectral flow cytometry - myeloid cells (C57BL/6, 4 litters)**	Sham Isotype early	1		
	Sham Isotype late	1	1	
	Sham Anti-Ly6G early		1	
	Sham Anti-Ly6G late		1	
	HI Isotype early	1	2	
	HI Isotype late	1	3	
	HI Anti-Ly6G early	4	3	1m*
	HI Anti-Ly6G late	3	3	

** died during/shortly after hypoxia, i.e. after randomization, but prior to treatment*

Table S3: Antibodies used for MACSima imaging

antigen	fluorophor	clone	vendor	catalog #	dilution
Ly6G	PE	1A8	Miltenyi Biotec	130-123-712	50
CD11b	APC	M1/70	Miltenyi Biotec	130-113-231	50
Siglec-F	PE	1RNM44N	eBioscience	12-1702-82	50
CD31	PE	polyclonal	R&D Bioscience	FAB3628P-100	50
MBP	FITC	REA1154	Miltenyi Biotec	130-120-341	50
Ki-67	APC	REA183	Miltenyi Biotec	130-120-416	50
NeuN	PE	REA1131	Miltenyi Biotec	130-119-493	50
CD86	APC	REA825	Miltenyi Biotec	130-112-857	50
CD11c	APC	REA754	Miltenyi Biotec	130-110-839	50

antigen	reactivity	host	vendor	catalog #	dilution
Laminin	mouse	rabbit	Sigma-Aldrich	L9393-100UL	100
rabbit IgG (FITC)	rabbit	goat	Sigma-Aldrich	F0382-1M	100

Table S4: Antibodies used for immunohistochemistry

antigen	reactivity	host	vendor	catalog #	dilution
NeuN	mouse	rabbit	Millipore	ABM78	500
CD31	mouse	rat	BD Biosciences	550274	100
Olig2	mouse	mouse	Millipore	MABN50	100
Olig2	mouse	rabbit	Millipore	AB9610	100
Ki67	mouse/rat	rabbit	Abcam	ab66155	250
CC1	mouse/rat	mouse	Calbiochem	OP80	100
DCX	mouse	mouse	Santa Cruz	sc-271390	50
GFAP	mouse	rat	Invitrogen	13-0300	500
Iba-1	mouse/rat	rabbit	Wako	019-19741	500

Table S5: TaqMan assays and primer sequences

gene	Taqman assay #
mbp	Mm01266402
cc1	Mm00545872
cnpase	Mm01306641
mag	Mm00487541
map2	Mm00485231
neun	Mm01248781

gene	forward	reverse
vegfa	CTGCTGTAACGATGAAGCCCTG	GCTGTAGGAAGCTCATCTCTCC
ang1	AACCGAGCCTACTCACAGTACG	GCATCCTTCGTGCTGAAATCGG
ang2	AACTCGCTCCTTCAGAAGCAGC	TTCCGCACAGTCTCTGAAGGTG
il-4	ATCATCGGCATTTTGAACGAGGTC	ACCTTGGAAGCCCTACAGACGA
rps9	AGGATTTCTTGGAGAGGCGGCT	CTGAGAGTCCAGGCGAACAATG

Table S6: Antibodies used for cell sorting and conventional flow cytometry

antigen	fluorophor	clone	vendor	catalog #	dilution
CD45	Alexa Fluor 700	30-F11	BD Biosciences	560510	200
CD11b	eF450	M1/70	ebiosciences	48-0112-82	200
Ly6G	PE	1A8	BD Biosciences	561104	800
Ly6G	APC	1A8	biolegend	127613	200
CD49d	Alexa Fluor 647	R1-2	BD Biosciences	564394	400
CXCR4	BV 421	L276F12	biolegend	146511	50
Siglec F	PE	E50-2440	BD Biosciences	552126	100
VEGFR-1	Fitc	141522	R&D Systems	FAB4711G	200

Table S7: Antibodies used for spectral flow cytometry – neutrophils

antigen	fluorophor	clone	vendor	catalog #	dilution
Ly6G	BUV395	1A8	BD Biosciences	565964	300
CD11b	BUV496	M1/70	BD Biosciences	749864	200
CD49d	BUV563	9C10	BD Biosciences	741243	100
MHC-II	BUV661	M5/114	BD Biosciences	750280	100
Siglec-F	BUV737	1RNM44N	Thermo Scientific	367-1702-82	100
CD117	BV421	2B8	BioLegend	105828	50
CD62L	BV480	MEL-14	BD Biosciences	746726	200
CD44	BV570	IM7	BioLegend	103037	300
CXCR4	BV711	L276F12	BioLegend	146517	50
Ly6C	BV785	HK1.4	BioLegend	128041	300
CD115	Alexa Fluor 488	AFS98	BioLegend	135512	100
CD45	PerCP	30-F11	BioLegend	103130	400
CXCR2	PE	SA044G4	BioLegend	149304	50
CD16	PE/Dazzle 594	S17014E	BioLegend	158012	200
CD80	PE-Cy7	16-10A1	BioLegend	104734	200
CD101	APC	Moushi101	Thermo Scientific	17-1011-82	100
CD24	Alexa Fluor 700	M1/69	BioLegend	101836	200
CD14	APC/Fire 750	Sa14-2	BioLegend	123332	200

Table S8: Antibodies used for spectral flow cytometry – myeloid cells

antigen	fluorophor	clone	vendor	catalog #	dilution
Ly6C	BV785	HK1.4	BioLegend	128041	300
CD11b	BUV496	M1/70	BD Biosciences	749864	200
CD45	PerCP	30-F11	BioLegend	103130	400
P2RY12	APC/Cy7	S16007D	Biolegend	848023	100
TMEM119	PerCP-eFluor 710	V3RT1GOsz	ThermoScientific	46-6119-80	100
CX3CR1	PE-Cy5	SA011F11	Biolegend	149049	100
CD11c	Alexa Fluor 488	N418	Biolegend	117313	100
CD115	BUV737	AFS98	BD Biosciences	750948	100
CD64	PE-Cy7	X54-5/7.1	Biolegend	139313	100
CCR2	BV750	475301	BD Biosciences	747967	100
TREM2	PE	6 E9	Biolegend	824805	100
C5AR1	APC	20/70	Biolegend	135807	100
CD206	eFluor 450	19 2	eBioscience	48-2069-42	100
CD169	BV605	3D6.112	BioLegend	142413	100
MHCII	BUV661	M5/114	BD Biosciences	750280	100

Table S9: Chemoattractants screened in Complex Eye assays

stimulus	vendor	catalog #	stock concentration	assay concentration
CXCL1	R&D Systems	275-GR/CF	10 µg/ml in PBS	0.2 µg / ml
CXCL2	R&D Systems	452-M2/CF	10 µg/ml in PBS	0.2 µg / ml
CXCL5	Biolegend	573302	10 µg/ml in PBS	0.2 µg / ml
CXCL12	Peprotech	250-20B-2UG	10 µg/ml in PBS	0.2 µg / ml
CXCL10	Peprotech	300-12-5ug	10 µg/ml in PBS	0.2 µg / ml
CXCL16	Peprotech	250-28-5UG	10 µg/ml in PBS	0.2 µg / ml
CCL6	Peprotech	250-06-2UG	10 µg/ml in PBS	0.2 µg / ml
CCLL11	Biolegend	583002	10 µg/ml in PBS	0.2 µg / ml
LTB4	R&D Systems	2307/50U	10 µg/ml in ethanol	0.2 µg / ml
PAF	Merck	511075	2 µg/ml in 0.2% ethanol	0.4 µg / ml
GM-CSF	Biolegend	576304	25 µg/ml in PBS	0.5 µg / ml
Osteopontin	Biozym	B763602	25 µg/ml in PBS	0.5 µg / ml
