# Supplementary material for: Hypoxic-ischemic brain injury in neonatal mice sequentially recruits neutrophils with dichotomous phenotype and function
Source: Nat Commun. 2025 Nov 3;16:9696. doi: 10.1038/s41467-025-65517-1 (PMC12583616; doi:10.1038/s41467-025-65517-1)
Supplement: Supplementary file 2 — Description of Supplementary Information [file 41467_2025_65517_MOESM2_ESM.docx]

**Description of Additional Supplementary Files**

**Supplementary Movie S1:** Light sheet microscopy of a cleared brain tissue reveals neutrophil infiltration into the injured ipsilateral brain hemisphere.Neutrophils from a CatchupIVM mice at day 7 after HI are displayed in red (as spots or raw signal), while FITCgelatin perfused vessels are visualized in blue
