# Supplementary material for: Hypoxic-ischemic brain injury in neonatal mice sequentially recruits neutrophils with dichotomous phenotype and function
Source: Nat Commun. 2025 Nov 3;16:9696. doi: 10.1038/s41467-025-65517-1 (PMC12583616; doi:10.1038/s41467-025-65517-1)
Supplement: Supplementary file 4 — Reporting Summary [file 41467_2025_65517_MOESM4_ESM.pdf]

nature portfolio

Corresponding author(s): Josephine Herz
Last updated by author(s): 24.09.25

Reporting Summary

Nature Portfolio wishes to improve the reproducibility of the work that we publish. This form provides structure for consistency and transparency in reporting. For further information on Nature Portfolio policies, see our Editorial Policies and the Editorial Policy Checklist.

Statistics

For all statistical analyses, confirm that the following items are present in the figure legend, table legend, main text, or Methods section.

n/a	Confirmed	
[ ]	[x]	The exact sample size (*n*) for each experimental group/condition, given as a discrete number and unit of measurement
[ ]	[x]	A statement on whether measurements were taken from distinct samples or whether the same sample was measured repeatedly
[ ]	[x]	The statistical test(s) used AND whether they are one- or two-sided<br>*Only common tests should be described solely by name; describe more complex techniques in the Methods section.*
[x]	[ ]	A description of all covariates tested
[ ]	[x]	A description of any assumptions or corrections, such as tests of normality and adjustment for multiple comparisons
[ ]	[x]	A full description of the statistical parameters including central tendency (e.g. means) or other basic estimates (e.g. regression coefficient) AND variation (e.g. standard deviation) or associated estimates of uncertainty (e.g. confidence intervals)
[ ]	[x]	For null hypothesis testing, the test statistic (e.g. *F*, *t*, *r*) with confidence intervals, effect sizes, degrees of freedom and *P* value noted<br>*Give P values as exact values whenever suitable.*
[x]	[ ]	For Bayesian analysis, information on the choice of priors and Markov chain Monte Carlo settings
[x]	[ ]	For hierarchical and complex designs, identification of the appropriate level for tests and full reporting of outcomes
[ ]	[x]	Estimates of effect sizes (e.g. Cohen's *d*, Pearson's *r*), indicating how they were calculated

Our web collection on statistics for biologists contains articles on many of the points above.

Software and code

Policy information about availability of computer code

Data collection	Blaze Ultramicroscope, Noldus Ethovision XT15 tracking system, NIS A1 Eclipse Ti confocal microscrope, ChemiDocXRS+ imaging system, FACS Aria cell sorter, AMG EVOS inverted digital phase contrast microscope, BD FACS LSRII, 5L-Cytek® Aurora, MACSima imaging system, Leica TCS SP8 confocal microscope with Multi Photon (MP) excitation, StepOnePlus Real Time PCR system, ComplexEye multilense microscope

April 2023

Data analysis	IMARIS (version 9/10)<br>Noldus Ethovision XT (version 15.0)<br>NIS Elements AR software (version 4.2)<br>ImageJ (1.53/1.54)<br>FACS Diva software (version 6.1.3)<br>FlowJo (version:10.10)<br>GraphPad Prism (version 9/10)<br>R<br>Leica Application Suite (LAS X, version 3.1.5.16308)<br>MACS iQ View (version 1.3.2<br>ImageLab (version 5.1)<br>VesselExpress (Spangenberg P, et al. Rapid and fully automated blood vasculature analysis in 3D light-sheet image volumes of different organs. Cell Rep Methods 3, 100436 (2023) doi: 10.1016/j.crmeth.2023.100436)<br>Napari platform in combination with the MMV_H4Tracks plugin (Cibir Z, et al. ComplexEye: a multi-lens array microscope for high-throughput embedded immune cell migration analysis. Nat Commun 14, 8103 (2023) doi: 10.1038/s41467-023-43765-3)

For manuscripts utilizing custom algorithms or software that are central to the research but not yet described in published literature, software must be made available to editors and reviewers. We strongly encourage code deposition in a community repository (e.g. GitHub). See the Nature Portfolio guidelines for submitting code & software for further information.

Data

Policy information about availability of data

All manuscripts must include a data availability statement. This statement should provide the following information, where applicable:

- Accession codes, unique identifiers, or web links for publicly available datasets
- A description of any restrictions on data availability
- For clinical datasets or third party data, please ensure that the statement adheres to our policy

The data supporting the findings of this study are available within the article and its Supplementary Figures. The source data underlying the main and supplementary figures and details on statistical analyses are provided in the source data file.

Research involving human participants, their data, or biological material

Policy information about studies with human participants or human data. See also policy information about sex, gender (identity/presentation), and sexual orientation and race, ethnicity and racism.

Reporting on sex and gender	n/a
Reporting on race, ethnicity, or other socially relevant groupings	n/a
Population characteristics	n/a
Recruitment	n/a
Ethics oversight	n/a

Note that full information on the approval of the study protocol must also be provided in the manuscript.

Field-specific reporting

Please select the one below that is the best fit for your research. If you are not sure, read the appropriate sections before making your selection.

[x] Life sciences [ ] Behavioural & social sciences [ ] Ecological, evolutionary & environmental sciences

For a reference copy of the document with all sections, see nature.com/documents/nr-reporting-summary-flat.pdf

Life sciences study design

All studies must disclose on these points even when the disclosure is negative.

Sample size	Animal numbers per group are listed in each figure legend and summarized (incl. mortality rates) in Suppl. Tabel S2.<br>Sample size for immunohistochemistry analyses and assessment of long-term functional deficits were determined a priori using G*Power, assuming and effect size f [by ANOVA] of 0.6, an α-level of 0.05 and a power of 0.8 were required. A mortality of 10% was assumed for the present injury model, yielding a final sample size of 10 animals per group. All other analyses were exploratory analyses.
Data exclusions	All animals were included in data analyses. In Fig. 4 L only mice with >2% Siglec-Fhigh neutrophils at d7 are displayed.<br>For proteome profiler array analyses (Fig. 2A,B S2A,C) values below background signal were excluded from analyses.

Replication	For each readout, data were derived from 2-5 independent experiments with up to 4 litters (5-10 animals/litter) / experiment. Data from in vitro co-culture assays were obtained from 2-10 independent experiments with sorted neutrophils derived from 4-12 animals / experiment. Two photon images provided in Fig. 1C and S1C were acquired from different field of views in 1-2 mice / group since this kind of analyses was only intended to confirm results obtained from light sheet microscopy and conventional immunohistochemistry analyses presented in Fig. 1B, 1D-F, S1D,E. Detailed animal allocations are shown in the supporting summary.
Randomization	For all analyses, animals per litter and experiment were randomly assigned to treatment groups before surgery and treatement. To control for the potential influence of weight and sex, a stratified randomization was performed followed by simple randomization within each block to assign pups to individual groups.
Blinding	Individuals involved in data collection and analysis knew the animals' designation but were blinded to group assignment.

Behavioural & social sciences study design

All studies must disclose on these points even when the disclosure is negative.

Study description	*Briefly describe the study type including whether data are quantitative, qualitative, or mixed-methods (e.g. qualitative cross-sectional, quantitative experimental, mixed-methods case study).*
Research sample	*State the research sample (e.g. Harvard university undergraduates, villagers in rural India) and provide relevant demographic information (e.g. age, sex) and indicate whether the sample is representative. Provide a rationale for the study sample chosen. For studies involving existing datasets, please describe the dataset and source.*
Sampling strategy	*Describe the sampling procedure (e.g. random, snowball, stratified, convenience). Describe the statistical methods that were used to predetermine sample size OR if no sample-size calculation was performed, describe how sample sizes were chosen and provide a rationale for why these sample sizes are sufficient. For qualitative data, please indicate whether data saturation was considered, and what criteria were used to decide that no further sampling was needed.*
Data collection	*Provide details about the data collection procedure, including the instruments or devices used to record the data (e.g. pen and paper, computer, eye tracker, video or audio equipment) whether anyone was present besides the participant(s) and the researcher, and whether the researcher was blind to experimental condition and/or the study hypothesis during data collection.*
Timing	*Indicate the start and stop dates of data collection. If there is a gap between collection periods, state the dates for each sample cohort.*
Data exclusions	*If no data were excluded from the analyses, state so OR if data were excluded, provide the exact number of exclusions and the rationale behind them, indicating whether exclusion criteria were pre-established.*
Non-participation	*State how many participants dropped out/declined participation and the reason(s) given OR provide response rate OR state that no participants dropped out/declined participation.*
Randomization	*If participants were not allocated into experimental groups, state so OR describe how participants were allocated to groups, and if allocation was not random, describe how covariates were controlled.*

Ecological, evolutionary & environmental sciences study design

All studies must disclose on these points even when the disclosure is negative.

Study description	*Briefly describe the study. For quantitative data include treatment factors and interactions, design structure (e.g. factorial, nested, hierarchical), nature and number of experimental units and replicates.*
Research sample	*Describe the research sample (e.g. a group of tagged Passer domesticus, all Stenocereus thurberi within Organ Pipe Cactus National Monument), and provide a rationale for the sample choice. When relevant, describe the organism taxa, source, sex, age range and any manipulations. State what population the sample is meant to represent when applicable. For studies involving existing datasets, describe the data and its source.*
Sampling strategy	*Note the sampling procedure. Describe the statistical methods that were used to predetermine sample size OR if no sample-size calculation was performed, describe how sample sizes were chosen and provide a rationale for why these sample sizes are sufficient.*
Data collection	*Describe the data collection procedure, including who recorded the data and how.*
Timing and spatial scale	*Indicate the start and stop dates of data collection, noting the frequency and periodicity of sampling and providing a rationale for these choices. If there is a gap between collection periods, state the dates for each sample cohort. Specify the spatial scale from which the data are taken*
Data exclusions	*If no data were excluded from the analyses, state so OR if data were excluded, describe the exclusions and the rationale behind them, indicating whether exclusion criteria were pre-established.*
Reproducibility	*Describe the measures taken to verify the reproducibility of experimental findings. For each experiment, note whether any attempts to repeat the experiment failed OR state that all attempts to repeat the experiment were successful.*

Randomization	*Describe how samples/organisms/participants were allocated into groups. If allocation was not random, describe how covariates were controlled. If this is not relevant to your study, explain why.*
Blinding	*Describe the extent of blinding used during data acquisition and analysis. If blinding was not possible, describe why OR explain why blinding was not relevant to your study.*

Did the study involve field work? ☐ Yes ☐ No

Field work, collection and transport

Field conditions	*Describe the study conditions for field work, providing relevant parameters (e.g. temperature, rainfall).*
Location	*State the location of the sampling or experiment, providing relevant parameters (e.g. latitude and longitude, elevation, water depth).*
Access & import/export	*Describe the efforts you have made to access habitats and to collect and import/export your samples in a responsible manner and in compliance with local, national and international laws, noting any permits that were obtained (give the name of the issuing authority, the date of issue, and any identifying information).*
Disturbance	*Describe any disturbance caused by the study and how it was minimized.*

Reporting for specific materials, systems and methods

We require information from authors about some types of materials, experimental systems and methods used in many studies. Here, indicate whether each material, system or method listed is relevant to your study. If you are not sure if a list item applies to your research, read the appropriate section before selecting a response.

Materials & experimental systems

n/a	Involved in the study	
☐	☒	Antibodies
☐	☒	Eukaryotic cell lines
☒	☐	Palaeontology and archaeology
☐	☒	Animals and other organisms
☒	☐	Clinical data
☒	☐	Dual use research of concern
☒	☐	Plants

Methods

n/a	Involved in the study	
☒	☐	ChIP-seq
☐	☒	Flow cytometry
☒	☐	MRI-based neuroimaging

Antibodies

Antibodies used

In vivo depletion:
anti-Ly6G (clone 1A8, BioXCell, 5 µg/g)
anti-rat kappa immunoglobulin (clone MAR 18.5, BioXCell, 5µg/g)
rat IgG2a (clone 2A3, BioXCell, 5µg/g)
Immunohistochemistry:
antigen/ reactivity/ host/ vendor/ catalog/ dilution
NeuN/ mouse/ rabbit/ Millipore/ ABM78/ 500
CD31/ mouse/ rat/ BD Biosciences/ 550274/ 100
Olig2/ mouse/ mouse/ Millipore/ MABN50/ 100
Olig2/ mouse/ rabbit/ Millipore/ AB9610/ 100
Ki67/ mouse&rat/ rabbit/ Abcam/ ab66155/ 250
CC1/ mouse&rat/ mouse/ Calbiochem/ OP80/ 100
GFAP/ mouse/ rat/ Invitrogen/ 13-0300/ 500
Iba-1/ mouse&rat/ rabbit/ Wako/ 019-19741/ 500
DCX/ mouse/ mouse/ Santa Cruz/ sc-271390 50
Conventional Flow Cytometry and Cell Sorting
antigen/ fluorophor/ clone/ vendor/ catalog/ dilution
CD45/ Alexa Fluor 700/ 30-F11/ BD Biosciences/ 560510/ 200
CD11b/ eF450/ M1/70/ ebiosciences/ 48-0112-82/ 200
Ly6G PE/ 1A8/ BD Biosciences/ 561104/ 800
CD49d/ Alexa Fluor 647/ R1-2/ BD Biosciences/ 564394/ 400
VEGFR-1 Fitc/ 141522/ R&D Systems/ FAB4711G/ 200
Ly6G/ APC/ 1A8/ biolegend/ 127613/ 200
CD49d/ Alexa Fluor 647/ R1-2/ BD Biosciences/ 564394/ 400

CXCR4/ BV 421/ L276F12/ biolegend/ 146511/ 50
Siglec F/ PE/ E50-2440/ BD Biosciences/ 552126/ 100
VEGFR-1/ Fitc/ 141522/ R&D Systems/ FAB4711G/ 200
Spectral Flow Cytometry neutrophils
antigen/ fluorophor/ clone/ vendor/ catalog/ dilution
Ly6G/ BUV395/ 1A8/ BD Biosciences/ 565964/ 300
CD11b/ BUV496/ M1/70/ BD Biosciences/ 749864/ 200
CD49d/ BUV563/ 9C10/ BD Biosciences/ 741243/ 100
MHC-II/ BUV661/ M5/114/ BD Biosciences/ 750280/ 100
Siglec-F/ BUV737/ 1RNM44N/ Thermo Fisher scientific/ 367-1702-82/ 100
CD117/ BV421/ 2B8/ BioLegend/ 105828/ 50
CD62L/ BV480/ MEL-14/ BD Biosciences/ 746726/ 200
CD44/ BV570/ IM7/ BioLegend/ 103037/ 300
CXCR4/ BV711/ L276F12/ BioLegend/ 146517/ 50
Ly6C/ BV785/ HK1.4/ BioLegend/ 128041/ 300
CD115/ Alexa Fluor 488/ AFS98/ BioLegend/ 135512/ 100
CD45/ PerCP/ 30-F11/ BioLegend/ 103130/ 400
CXCR2/ PE/ SA044G4/ BioLegend/ 149304/ 50
CD16/ PE/Dazzle 594/ S17014E/ BioLegend/ 158012/ 200
CD80/ PE-Cy7/ 16-10A1/ BioLegend/ 104734/ 200
CD101/ APC/ Moushi101/ Thermo Fisher scientific/ 17-1011-82/ 100
CD24/ Alexa Fluor 700/ M1/69/ BioLegend/ 101836/ 200
CD14/ APC/Fire 750/ Sa14-2/ BioLegend/ 123332/ 200
Spectral Flow Cytometry myeloid cells
antigen/ fluorophor/ clone/ vendor/ catalog/ dilution
Ly6C/ BV785/ HK1.4/ BioLegend/ 128041/ 300
CD11b/ BUV496/ M1/70/ BD Biosciences/ 749864/ 200
CD45/ PerCP/ 30-F11/ BioLegend/ 103130/ 400
P2RY12/ APC/Cy7/ S16007D / Biolegend/ 848023/ 100
TMEM119/ PerCP-eFluor 710/ V3RT1GOsz/ ThermoFisher/ 46-6119-80/ 100
CX3CR1/ PE-Cy5/ SA011F11/ Biolegend/ 149049/ 100
CD11c/ Alexa Fluor 488/ N418/ Biolegend/ 117313/ 100
CD115/ BUV737/ AFS98/ BD Biosciences/ 750948/ 100
CD64/ PE-Cy7/ X54-5/7.1/ Biolegend/ 139313/ 100
CCR2/ BV750/ 475301/ BD Biosciences/ 747967/ 100
TREM2/ PE/ 6E9/ Biolegend/ 824805/ 100
C5AR1/ APC/ 20/70/ Biolegend/ 135807/ 100
CD206/ eFluor 450/ 19.2/ eBioscience/ 48-2069-42/ 100
CD169/ BV605/ 3D6.112/ BioLegend/ 142413/ 100
MHC-II/ BUV661/ M5/114/ BD Biosciences/ 750280/ 100
MACSima
antigen/ fluorophor/ clone/ vendor/ catalog/ dilution
Ly6G/ PE/ 1A8/ Miltenyi Biotec/ 130-123-712/ 50
CD11b/ APC/ M1/70/ Miltenyi Biotec/ 130-113-231/ 50
Siglec-F/ PE/ 1RNM44N/ eBioscience/ 12-1702-82/ 50
CD31/ PE/ polyclonal/ R&D Bioscience/ FAB3628P-100 / 50
MBP/ FITC/ REA1154/ Miltenyi Biotec/ 130-120-341/ 50
Ki-67/ APC/ REA183/ Miltenyi Biotec/ 130-120-416/ 50
NeuN/ PE/ REA1131/ Miltenyi Biotec/ 130-119-493/ 50
CD68/ APC/ REA835/ Miltenyi Biotec/ 130-112-857/ 50
CD11c/ APC/ REA754/ Miltenyi Biotec/ 130-110-839/ 50
antigen/ reactivity/ host/ vendor/ catalog/ dilution
laminin/ mouse/ rabbit/ Sigma-Aldrich/ L9393-100UL/ 100
FITC anti-rabbit IgG/ rabbit / goat/ Sigma-Aldrich/ F0382-1ML/ 100

Validation

Antibody concentrations were determined in titration tests of single staining and further tested in multicolor staining. Staining with secondary antibodies served as controls in immunohistochemistry analyses. FMO controls were used in flow cytometry analyses. All antibodies were bought from commercial suppliers and validations are available by the manufacturers on their respective websites using catalog number searches.

Eukaryotic cell lines

Policy information about cell lines and Sex and Gender in Research

Cell line source(s)

mouse brain microcascular endothelial cells (bEnd.3 Catalog CRL-2299 (ATCC)

Authentication

This cell line is not listed as a misidentified cell line in the ICLAC register. It was not authenticated

Mycoplasma contamination

Cells were not tested for mycoplasma

Commonly misidentified lines (See ICLAC register)	*Name any commonly misidentified cell lines used in the study and provide a rationale for their use.*

Palaeontology and Archaeology

Specimen provenance	*Provide provenance information for specimens and describe permits that were obtained for the work (including the name of the issuing authority, the date of issue, and any identifying information). Permits should encompass collection and, where applicable, export.*
Specimen deposition	*Indicate where the specimens have been deposited to permit free access by other researchers.*
Dating methods	*If new dates are provided, describe how they were obtained (e.g. collection, storage, sample pretreatment and measurement), where they were obtained (i.e. lab name), the calibration program and the protocol for quality assurance OR state that no new dates are provided.*

☐ Tick this box to confirm that the raw and calibrated dates are available in the paper or in Supplementary Information.

Ethics oversight	*Identify the organization(s) that approved or provided guidance on the study protocol, OR state that no ethical approval or guidance was required and explain why not.*

Note that full information on the approval of the study protocol must also be provided in the manuscript.

Animals and other research organisms

Policy information about studies involving animals; ARRIVE guidelines recommended for reporting animal research, and Sex and Gender in Research

Laboratory animals	C57BL/6JOlaHsd mice were initially obtained from Envigo (Netherlands) followed by in house breeding. CatchupIVM mice were recently developed in the laboratory of M. Gunzer, followed by in house breeding. Mice were housed in specific pathogen free individually ventilated cages, kept under a 12-h light/dark cycle with food and water ad libitum. Room temperature and relative humidity were maintained between 20–24 °C and 45–65%, respectively. Nine day old mice were used.
Wild animals	This study did not involve wild animals.
Reporting on sex	Female and male animals per litter were equally assigned to treatment groups. The present study was not intended to determine sex differences. All data are reported disaggregated for sex in the source data file.
Field-collected samples	This study did not involve samples collected from the field
Ethics oversight	Experiments were performed in accordance with the Animal Research Reporting of in Vivo Experiments (ARRIVE) guidelines with governmental approval by the State Agency for Nature, Environment and Consumer Protection North Rhine-Westphalia, under permission numbers G1773/20 and G1778/20.

Note that full information on the approval of the study protocol must also be provided in the manuscript.

Clinical data

Policy information about clinical studies

All manuscripts should comply with the ICMJE guidelines for publication of clinical research and a completed CONSORT checklist must be included with all submissions.

Clinical trial registration	*Provide the trial registration number from ClinicalTrials.gov or an equivalent agency.*
Study protocol	*Note where the full trial protocol can be accessed OR if not available, explain why.*
Data collection	*Describe the settings and locales of data collection, noting the time periods of recruitment and data collection.*
Outcomes	*Describe how you pre-defined primary and secondary outcome measures and how you assessed these measures.*

Dual use research of concern

Policy information about dual use research of concern

Hazards

Could the accidental, deliberate or reckless misuse of agents or technologies generated in the work, or the application of information presented in the manuscript, pose a threat to:

No	Yes	
☐	☐	Public health
☐	☐	National security
☐	☐	Crops and/or livestock
☐	☐	Ecosystems
☐	☐	Any other significant area

Experiments of concern

Does the work involve any of these experiments of concern:

No	Yes	
☐	☐	Demonstrate how to render a vaccine ineffective
☐	☐	Confer resistance to therapeutically useful antibiotics or antiviral agents
☐	☐	Enhance the virulence of a pathogen or render a nonpathogen virulent
☐	☐	Increase transmissibility of a pathogen
☐	☐	Alter the host range of a pathogen
☐	☐	Enable evasion of diagnostic/detection modalities
☐	☐	Enable the weaponization of a biological agent or toxin
☐	☐	Any other potentially harmful combination of experiments and agents

Plants

Seed stocks	n/a
Novel plant genotypes	n/a
Authentication	n/a

ChIP-seq

Data deposition

☐ Confirm that both raw and final processed data have been deposited in a public database such as GEO.

☐ Confirm that you have deposited or provided access to graph files (e.g. BED files) for the called peaks.

Data access links<br>*May remain private before publication.*	*For "Initial submission" or "Revised version" documents, provide reviewer access links. For your "Final submission" document, provide a link to the deposited data.*
Files in database submission	*Provide a list of all files available in the database submission.*
Genome browser session<br>(e.g. UCSC)	*Provide a link to an anonymized genome browser session for "Initial submission" and "Revised version" documents only, to enable peer review. Write "no longer applicable" for "Final submission" documents.*

Methodology

Replicates	*Describe the experimental replicates, specifying number, type and replicate agreement.*
Sequencing depth	*Describe the sequencing depth for each experiment, providing the total number of reads, uniquely mapped reads, length of reads and whether they were paired- or single-end.*
Antibodies	*Describe the antibodies used for the ChIP-seq experiments; as applicable, provide supplier name, catalog number, clone name, and lot number.*
Peak calling parameters	*Specify the command line program and parameters used for read mapping and peak calling, including the ChIP, control and index files used.*
Data quality	*Describe the methods used to ensure data quality in full detail, including how many peaks are at FDR 5% and above 5-fold enrichment.*

Software | *Describe the software used to collect and analyze the ChIP-seq data. For custom code that has been deposited into a community repository, provide accession details.*

Flow Cytometry

Plots

Confirm that:

- [x] The axis labels state the marker and fluorochrome used (e.g. CD4-FITC).
- [ ] The axis scales are clearly visible. Include numbers along axes only for bottom left plot of group (a 'group' is an analysis of identical markers).
- [x] All plots are contour plots with outliers or pseudocolor plots.
- [x] A numerical value for number of cells or percentage (with statistics) is provided.

Methodology

Sample preparation | Blood specimens were collected with ethylenediaminetetraacetate (EDTA) coated capillaries (CLINITUBES, Radiometer, Germany) by snipping the right atrium of the heart immediately prior to perfusion via the left ventricle and transferred into EDTA coated collection tubes (Minicollect, Greiner Bio One, Germany). Ipsilateral brain hemispheres and spleens were dissected and homogenized through a 70 µm cell strainer (BD Biosciences) by continuous rinsing with 15 ml of ice-cold hanks buffered saline solution (HBSS, Gibco, Thermo Scientific) supplemented with 0.06% bovine serum albumin (Sigma-Aldrich), 0.6 mM EDTA (Carl Roth, Germany), pH 7.4. Erythrocytes in spleen and blood samples were lysed by incubation with RBC lysis buffer (BioLegend, USA) for 1 min (spleen) and 5 min (blood) on ice followed by a washing step with HBSS. Neonatal femurs and tibiae were pre-shredded before the bone marrow was homogenized through a 70 µm cell strainer by continuous rinsing with 15 ml of ice-cold HBSS. Homogenized brain samples were centrifuged at 400×g for 10 min at 18°C and the supernatant was discarded. The pellets were resuspended in 7 ml 37% Percoll (Sigma Aldrich, Germany) in 0.01 N HCl/PBS and centrifuged at 2800×g for 20 min at 18°C. The cell pellet was washed in HBSS prior to staining in Cell Staining Buffer (BioLegend) or HBSS.

Instrument | BD FACS Aria, BD FACS LSRII, 5L-Cytek® Aurora

Software | BD FACS Diva, FlowJo

Cell population abundance | neutrophils, myeloid subsets

Gating strategy | To identify neutrophils in conventional/spectral flow cytometry and for sorting experiments, doublets were excluded using scatter characteristics and viable cells were identified via staining for fixable viability dye (FvD) or Dapi. Specific gating strategies are provided in main and Suppl. Figures of the manuscript.

- [x] Tick this box to confirm that a figure exemplifying the gating strategy is provided in the Supplementary Information.

Magnetic resonance imaging

Experimental design

Design type | *Indicate task or resting state; event-related or block design.*

Design specifications | *Specify the number of blocks, trials or experimental units per session and/or subject, and specify the length of each trial or block (if trials are blocked) and interval between trials.*

Behavioral performance measures | *State number and/or type of variables recorded (e.g. correct button press, response time) and what statistics were used to establish that the subjects were performing the task as expected (e.g. mean, range, and/or standard deviation across subjects).*

Acquisition

Imaging type(s) | *Specify: functional, structural, diffusion, perfusion.*

Field strength | *Specify in Tesla*

Sequence & imaging parameters | *Specify the pulse sequence type (gradient echo, spin echo, etc.), imaging type (EPI, spiral, etc.), field of view, matrix size, slice thickness, orientation and TE/TR/flip angle.*

Area of acquisition | *State whether a whole brain scan was used OR define the area of acquisition, describing how the region was determined.*

Diffusion MRI [ ] Used [ ] Not used

Preprocessing

Preprocessing software | *Provide detail on software version and revision number and on specific parameters (model/functions, brain extraction, segmentation, smoothing kernel size, etc.).*

Normalization	*If data were normalized/standardized, describe the approach(es): specify linear or non-linear and define image types used for transformation OR indicate that data were not normalized and explain rationale for lack of normalization.*
Normalization template	*Describe the template used for normalization/transformation, specifying subject space or group standardized space (e.g. original Talairach, MNI305, ICBM152) OR indicate that the data were not normalized.*
Noise and artifact removal	*Describe your procedure(s) for artifact and structured noise removal, specifying motion parameters, tissue signals and physiological signals (heart rate, respiration).*
Volume censoring	*Define your software and/or method and criteria for volume censoring, and state the extent of such censoring.*

Statistical modeling & inference

Model type and settings	*Specify type (mass univariate, multivariate, RSA, predictive, etc.) and describe essential details of the model at the first and second levels (e.g. fixed, random or mixed effects; drift or auto-correlation).*
Effect(s) tested	*Define precise effect in terms of the task or stimulus conditions instead of psychological concepts and indicate whether ANOVA or factorial designs were used.*

Specify type of analysis: ☐ Whole brain ☐ ROI-based ☐ Both

Statistic type for inference (See Eklund et al. 2016)	*Specify voxel-wise or cluster-wise and report all relevant parameters for cluster-wise methods.*
Correction	*Describe the type of correction and how it is obtained for multiple comparisons (e.g. FWE, FDR, permutation or Monte Carlo).*

Models & analysis

n/a	Involved in the study
☐	☐ Functional and/or effective connectivity
☐	☐ Graph analysis
☐	☐ Multivariate modeling or predictive analysis

Functional and/or effective connectivity	*Report the measures of dependence used and the model details (e.g. Pearson correlation, partial correlation, mutual information).*
Graph analysis	*Report the dependent variable and connectivity measure, specifying weighted graph or binarized graph, subject- or group-level, and the global and/or node summaries used (e.g. clustering coefficient, efficiency, etc.).*
Multivariate modeling and predictive analysis	*Specify independent variables, features extraction and dimension reduction, model, training and evaluation metrics.*
